# Severe outcomes among adults with TB during COVID-19

**DOI:** 10.5588/ijtldopen.24.0220

**Published:** 2024-07-01

**Authors:** T. Jacobs, E. Morden, M. Smith, A. von Delft, R. Kassanjee, V. Mudaly, A. Boulle, M-A. Davies

**Affiliations:** ^1^ Health Intelligence Directorate, Western Cape Health and Wellness, Provincial Government of the Western Cape, Cape Town, South Africa;; ^2^ School of Public Health, University of Cape Town, Cape Town, South Africa;; ^3^ Centre for Infectious Disease Epidemiology & Research, School of Public Health, University of Cape Town, South Africa;; ^4^ Western Cape Health and Wellness, Provincial Government of the Western Cape, Cape Town, South Africa

**Keywords:** TB control, pandemic impact, outcome

## Abstract

**BACKGROUND:**

The COVID-19 pandemic prompted strict public health measures to reduce SARS-CoV-2 transmission, potentially interrupting TB programmes in the Western Cape, South Africa.

**METHODS:**

We conducted a retrospective cohort study, estimating changes in new TB case rates and risk of death during TB-specific admissions within 6 months of TB first evidence, during the pre-pandemic (1 January 2019–26 March 2020) and after the implementation of public health and social measures (PHSM) periods (26 March 2020–30 September 2021), based on PHSM strictness. We used interrupted time series and logistic regression models to adjust for key characteristics.

**RESULTS:**

We found an average 22% reduction (95% CI 19–25) in monthly TB cases during the entire PHSM implementation period. Additionally, the risk of death during TB-specific admissions increased, with the adjusted odds ratio ranging across PHSM levels from 1.36 (95% CI 1.17–1.57) on Level 1 to 1.44 (95% CI 1.16–1.79) on Level 2 compared with the pre-pandemic period.

**CONCLUSIONS:**

There was a decline in the number of diagnosed TB cases and an increased risk of severe outcomes from 26 March 2020 to 30 September 2021 in the Western Cape. TB programme recovery strategies must be prioritised, and TB management programmes must be integrated into future pandemic responses.

## RÉSUMÉ

**CONTEXTE:**

La pandémie de COVID-19 a entraîné la mise en place de mesures de santé publique strictes afin de limiter la propagation du SRAS-CoV-2, ce qui risque de perturber les efforts de lutte contre la TB dans la province du Cap-Occidental, en Afrique du Sud.

**MÉTHODES:**

Une étude de cohorte rétrospective a été réalisée afin d'évaluer les variations des taux de nouveaux cas de TB et le risque de décès lors des admissions spécifiques à la TB dans les 6 mois suivant la première preuve de la maladie. Cette étude a été menée pendant deux périodes distinctes : la période prépandémique, allant du 1er janvier 2019 au 26 mars 2020, et la période post-mise en œuvre des mesures de santé publique et sociales (PHSM, pour l’anglais « *public health and social measures* »), allant du 26 mars 2020 au 30 septembre 2021. L'objectif était d'évaluer l'impact des PHSM sur ces indicateurs. Pour ce faire, des séries temporelles interrompues et des modèles de régression logistique ont été utilisés afin de prendre en compte les principales caractéristiques.

**RÉSULTATS:**

Au cours de la période de mise en œuvre des PHSM, nous avons observé une diminution moyenne de 22% (IC à 95% 19–25) des cas mensuels de TB. De plus, nous avons constaté une augmentation du risque de décès pendant les admissions pour TB, avec un rapport de cotes ajusté variant de 1,36 (IC à 95% ; 1,17–1,57) au niveau 1 des PHSM à 1,44 (IC à 95% ; 1,16–1,79) au niveau 2, par rapport à la période prépandémique.

**CONCLUSIONS:**

Entre le 26 mars 2020 et le 30 septembre 2021, il y a eu une baisse du nombre de cas de TB diagnostiqués dans la province du Cap-Occidental, mais le risque de résultats graves a augmenté pendant cette période. Les stratégies de rétablissement des programmes de lutte contre la TB doivent être prioritaires et les programmes de gestion de la TB doivent être intégrés dans les futures réponses à la pandémie.

Globally, TB is one of the leading causes of death from a single infectious agent.^1,2^ South Africa has a high prevalence of TB, with an estimated 852 TB cases per 100,000 population (95% confidence interval [CI] 679–1,026) aged ≥15 years in 2018.^3^ The Western Cape Government Tuberculosis dashboard shows that between 1 January 2015 and 31 December 2021, a median of 50,095 TB cases were diagnosed annually in the province with a median of 4,248 annual deaths.^4^

With the emergence of the first known COVID-19 case in South Africa in early March 2020, several public health and social measures (PHSM) were implemented to reduce SARS-CoV-2 transmission, manage case load, and protect the public health service platform. While some of these measures may have reduced TB transmission within communities,^5^ there is uncertainty regarding the overall impact of restrictions, including movement restrictions, socio-economic consequences, and reduced access to services on TB and HIV programmes in resource-constrained regions.

In 2020, the South African National Institute for Communicable Diseases (NICD) noted a decrease in national Xpert^®^ MTB/RIF (GXP; Cepheid, Sunnyvale, CA, USA) nucleic acid amplification testing rates.^6^ By the end of 2020, TB testing in South Africa had decreased by 22% overall compared to 2019,^7^ with a 26% reduction in notified cases,^7^ which aligns with decreases in other high-burden countries.^8–10^ In the Western Cape, median monthly GXP tests decreased by 16.9% to 16,888 (April 2020–September 2021) from 20,327 tests (January 2019–March 2020).^4^ With such a substantial reduction in TB testing, there is concern that TB diagnoses may have been delayed and/or restricted to those with more severe disease, with consequent worse outcomes in those diagnosed. Using routine individual data from people using public sector health services in the Western Cape, we describe and compare the incidence of new TB cases and changes in mortality risk among adults aged 18 years. Our analysis spans from the onset of the COVID-19 pandemic until the conclusion of strict PHSM alert levels in South Africa (26 March 2020–30 September 2021), comparing these findings to pre-pandemic data (1 January 2019–25 March 2020).

## METHODS

This retrospective cohort study used de-identified data from routinely collected sources collated by the Western Cape Provincial Health Data Centre (WCPHDC). The WCPHDC is a health information exchange system used for clinical care by the Western Cape Government Department of Health and Wellness (WCGHW).^11,12^ The WCPHDC uses evidence from multiple sources to infer health conditions (called ‘episodes’). TB episodes were inferred from laboratory test results, TB registers, International Classification of Disease, Tenth Revision (ICD-10) codes, and drugs dispensed. Treatment outcomes were based on registry entries.^13^

The WCPHDC was used to populate an enumerated dataset of adults diagnosed with TB during the study period, as well as treatment information, comorbidities, and vital status. Deaths in the dataset encompass all causes, necessitating additional constraints to establish a robust link to the relevant TB episode. The primary outcome of interest was death during TB-specific admission. Admissions were considered TB-specific if the admission occurred within 6 months (182 days) after the first evidence date of TB and the primary diagnostic ICD-10 codes during admission were TB-related. Admissions with COVID-19-relevant ICD-10 codes or positive COVID-19 tests within 21 days before and 15 days after the admission date were not considered TB-specific. Deaths during TB-specific admission were included if the date of death was during hospitalisation for a TB-specific admission.

Because we do not have data on the cause of death, we are more confident that the deaths defined above were linked to TB because of sufficient evidence that the individual had active TB at the time of death and was primarily linked to TB during admission, with no evidence of concurrent COVID-19. The secondary outcome was all-cause death within 6 months of the TB's first evidence date. It is less certain that these deaths were due to TB because COVID-19 co-infection may have been underdetermined for deaths outside the hospital during this period.

PHSM were instituted on 26 March 2020 in South Africa, with stricter measures (higher ‘alert levels’) implemented during COVID-19 waves when cases were highest and relaxed (lower ‘alert levels’) between waves (Table 1).^14^ The implemented measures for different alert levels involved varying degrees of limiting social interaction through curfews, closure of social and commercial institutions, travel restrictions, and mask-wearing in public. The study period was restricted to the end of Level 2 after COVID-19 Wave 3 on 30 September 2021. After this, measures remained at Level 1 despite the advent of Wave 4, and all restrictions were lifted on 5 April 2022.^14^ TB episodes and deaths were linked to the relevant PHSM alert level based on the TB first evidence date.

**Table 1.** Public health and social measures, 26 March 2020–30 September 2021.

Restrictions	Alert Level 5	Alert Level 4	Alert Level 3	Alert Level 2	Alert Level 1
Curfew	All day except for essential services and goods	Confined to 21:00–04:00 except for essential services	Confined to 22:00–04:00 except for essential services	Confined to 23:00–04:00 except if granted permission	No curfew
Educational institutions	Closed	Closed	Open, with social distancing measures	Open, with social distancing measures	Open, with social distancing measures
Wearing face masks/coverings	In public spaces	In public spaces	In public spaces	In public spaces	In public spaces
Travel	No travel between provinces, international borders closed	No travel between provinces, international borders closed	Travel between provinces allowed, international borders partially open	Travel between provinces allowed, international borders partially open	No restrictions
Retail shops and malls	Closed, except for essential goods	Open, with capacity limitations	Open, with capacity limitations	Open, safety measures to be in place	Open, safety measures to be in place
Social gatherings	Prohibited	Prohibited, capacity limitations at funerals	Strict limitations on social gatherings	Limitations on social gatherings	Proof of vaccination/negative COVID-19 test for social gatherings
Public transport	Restricted capacity, only for rendering essential services	Capacity limitations implemented	Capacity limitations implemented	Capacity limitations implemented	Capacity limitations implemented

* Dates Implemented: Alert Level 5 (26 March–30 April 2020); Alert Level 4 (1 May–31 May 2020, 28 June–25 July 2021); Alert Level 3 (1 June–17 August 2020, 29 December 2020–28 February 2021, 16–27 June 2021, 26 July–12 September 2021); Alert Level 2 (18 August 2020–20 September 2020, 31 May–15 June 2021, 13–30 September 2021); Alert Level 1 (21 September–28 December 2020, 1 March–30 May 2021).

We describe the trends of TB episodes, all-cause deaths, and deaths during TB-specific admissions counts relative to the PHSM alert level at the time when TB first evidence occurred and use a structural Bayesian interrupted time series model^15^ to compare counts before and after the advent of the COVID-19 pandemic.

Random intercept multivariable logistic regression models were used to describe changes in the risk of death during the various PHSM levels compared with those before the COVID-19 pandemic, adjusted for age, previous TB episodes, drug resistance, HIV and antiretroviral therapy (ART) status, SARS-CoV-2 infections during the episode, diabetes mellitus, TB first evidence month, and health district. A Bayesian aggregated binomial model adjusted for the same covariates described the interaction between HIV and ART status, PHSM levels, and death risk during TB-specific admissions.

All analyses were performed using R v4.3.2 (R Computing, Vienna, Austria). This study was approved by the University of Cape Town Health Research Ethics Committee, Cape Town, South Africa (HREC 257/2023) and WCGHW, Cape Town, South Africa. The requirement for individual informed consent was waived for this secondary analysis of the de-identified data.

## RESULTS

The dataset included 107,573 TB episodes during the study period. Three hundred ninety-four episodes did not meet the inclusion criteria. The final dataset included 107,179 TB episodes, 7,537 deaths, and 1,673 deaths during TB-specific admission within 6 months of the first evidence date.

**Figure 1.**
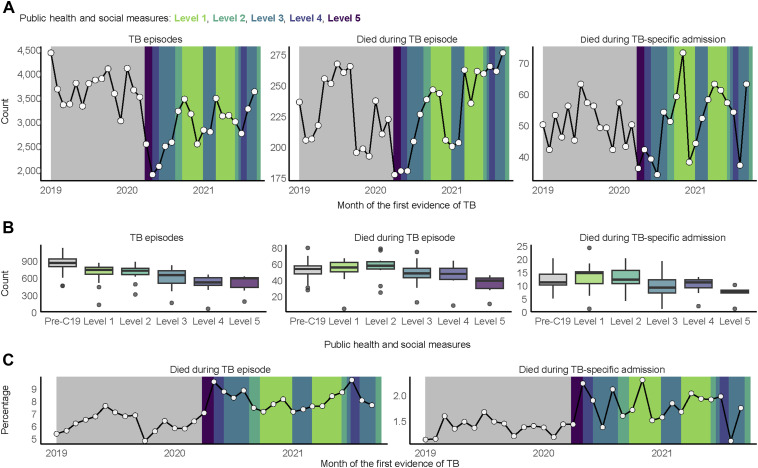
Changes in TB counts and percentages by public health and social measures. **A)** Monthly counts of TB episodes and deaths within 6 months from the first evidence of TB. **B)** Median weekly counts of TB episodes and deaths within 6 months after the first evidence of TB. **C)** Percentage of TB episodes with death within 6 months after the first evidence of TB.

The monthly count of diagnosed TB episodes (Figure 1A) was lowest during the early phase of the COVID-19 pandemic, particularly during high PHSM alert levels and in months before transition to lower alert levels (1,893 episodes, Level 4 in May 2020; 2,066 episodes, Level 3 in June 2020; and 2,482 episodes, Level 3 in July 2020). In general, monthly episode counts increased immediately after restrictions were relaxed to lower alert levels, with the highest counts in October 2020 (3,462 episodes, Level 1), March 2021 (3,477 episodes, Level 1), and September 2021 (3,617 episodes, Level 2).

In keeping with the lower number of TB episodes, all-cause deaths within 6 months after the first TB evidence had the lowest monthly count during Level 5 in April 2020 (177 deaths, 7% of episodes; Figure 1C) and May 2020 (180 deaths, 9.5% of episodes), with a subsequent increase as restrictions were relaxed to lower alert levels (246 deaths, 7.1% of episodes, Level 1 in October 2020; 262 deaths, 7.5% of episodes, Level 1 in March 2021). All-cause deaths were highest during July 2021 despite restrictions (265 deaths, 9.6% of episodes, Level 4), with the highest count for the entire study period in September 2021 (276 deaths, 7.6% of episodes) as restrictions were relaxed to Level 2. Deaths during TB-specific admissions followed a consistent pattern of increase immediately after the lowering of alert levels.

In total, 22% of deaths during TB-specific admissions occurred during Level 1 (Table 2). HIV and ART status are important factors among deaths during TB-specific admissions across the study period, with 48% of deaths occurring among those who are HIV positive and on ART during the episode (vs. 39% of those who survived the episode) and 17% of deaths among those who are positive but not on ART during the episode (vs. 4.1% of those who survived); 5% of diagnosed episodes had drug-resistant TB, and 11% of deaths during TB-specific admissions had drug-resistant TB across the study period.

**Table 2.** Summary of TB episodes, deaths during TB episodes and TB-specific admissions, 1 January 2019–30 September 2021.

Characteristic	TB episodes	Died during TB episode	Died during TB-specific admission
	(*n* = 107,179)	(*n* = 7,537)	(*n* = 1,673)
	*n* (%)	*n* (%)	*n* (%)
Public health and social measures			
Pre-COVID-19	54,961 (51)	3,392 (45)	753 (45)
Level 1	19,510 (18)	1,500 (20)	365 (22)
Level 2	7,470 (7.0)	610 (8.1)	140 (8.4)
Level 3	17,975 (17)	1,427 (19)	287 (17)
Level 4	4,332 (4.0)	407 (5.4)	87 (5.2)
Level 5	2,931 (2.7)	201 (2.7)	41 (2.5)
Age category, years			
18–29	28,680 (27)	915 (12)	258 (15)
30–44	44,631 (42)	2,794 (37)	696 (42)
45–59	25,449 (24)	2,310 (31)	473 (28)
≥60	8,419 (7.9)	1,518 (20)	246 (15)
Previous episodes			
0	74,531 (70)	4,855 (64)	1,039 (62)
1	22,357 (21)	1,710 (23)	423 (25)
2	7,200 (6.7)	646 (8.6)	129 (7.7)
>3	3,091 (2.9)	326 (4.3)	82 (4.9)
Drug-resistant status			
Drug-susceptible	101,854 (95)	6,965 (92)	1,489 (89)
Drug-resistant	5,325 (5.0)	572 (7.6)	184 (11)
HIV status during the episode			
HIV-negative	43,900 (41)	2,267 (30)	400 (24)
HIV-positive, not on ART	4,441 (4.1)	984 (13)	284 (17)
HIV-positive, on ART	42,281 (39)	3,248 (43)	809 (48)
HIV status unknown	16,557 (15)	1,038 (14)	180 (11)
COVID-19 during episode			
No infection	104,449 (97)	7,269 (96)	1,655 (99)
Infection	2,730 (2.5)	268 (3.6)	18 (1.1)
Diabetes			
Negative	100,161 (93)	6,632 (88)	1,533 (92)
Positive	7,018 (6.5)	905 (12)	140 (8.4)

ART = antiretroviral therapy.

**Figure 2.**
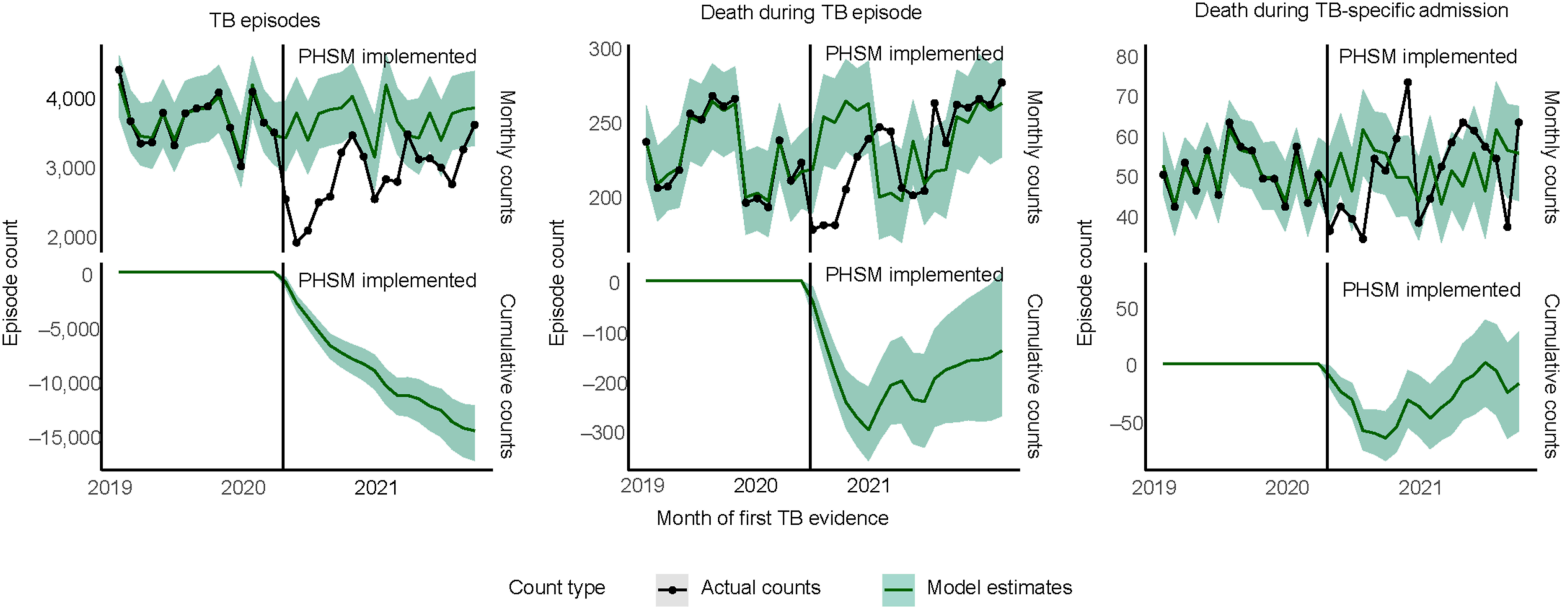
Interrupted time series models of TB episodes and deaths within 6 months after first evidence. PHSM = public health and social measures.

According to the interrupted time series model, the average monthly TB episode count was 22% lower (95% CI 19–25) from April 2020 to September 2021 compared with the model forecasted monthly average counterfactual count of 3,683 (95% CI 3,552–3,833) if there had been no pandemic or related effects (Figure 2). At the end of the study period, the cumulative difference between the actual and counterfactual episode counts was 14,473 episodes less than in which COVID-19 pandemic-related effects had not occurred (95% CI 12,130–17,178).

The effect on all-cause deaths within 6 months was more modest, with a relative decrease of 3.2% (95% CI 0.4–6.2; *P* = 0.03) compared with the counterfactual count. For the monthly count of deaths during TB-specific admissions, there was no statistical difference between the actual and counterfactual counts from April 2020 to September 2021.

The adjusted odds ratios (aORs) indicated that the risk of all-cause deaths within 6 months of the first TB evidence date (Table 3) was higher with all PHSM alert levels compared with pre-pandemic (January 2019–March 2020), except for Level 5, which had no significant difference, with the highest risk during Level 4 (aOR 1.4, 95% CI 1.2–1.5).

**Table 3.** Random intercept logistic regression models of TB deaths within 6 months from the first evidence.

Characteristic	Multivariable: death during TB-specific admission		Univariable: death during TB-specific admission		Multivariable: death during TB episode	
	aOR (95%CI)^*^	*P*-value	OR (95%CI)*	*P*-value	aOR (95%CI)*	*P*-value
Public health and social measures						
Pre-COVID-19	—		—		—	
Level 1	1.36 (1.17–1.57)	<0.001	1.37 (1.21–1.55)	<0.001	1.28 (1.19–1.38)	<0.001
Level 2	1.44 (1.16–1.79)	0.001	1.38 (1.15–1.65)	<0.001	1.26 (1.13–1.40)	<0.001
Level 3	1.18 (1.01–1.37)	0.040	1.16 (1.01–1.33)	0.031	1.21 (1.12–1.30)	<0.001
Level 4	1.34 (1.04–1.73)	0.023	1.48 (1.18–1.85)	<0.001	1.36 (1.20–1.54)	<0.001
Level 5	0.96 (0.67–1.37)	0.8	1.02 (0.75–1.41)	0.9	1.11 (0.93–1.32)	0.3
Age category, years						
18–29	—		—		—	
30–44	1.34 (1.15–1.55)	<0.001	1.76 (1.52–2.03)	<0.001	1.64 (1.51–1.77)	<0.001
45–59	1.85 (1.58–2.16)	<0.001	2.11 (1.81–2.46)	<0.001	2.70 (2.49–2.93)	<0.001
≥60	4.01 (3.33–4.83)	<0.001	3.36 (2.81–4.00)	<0.001	7.31 (6.67–8.00)	<0.001
Previous episodes						
0	—		—		—	
1	1.25 (1.11–1.41)	<0.001	1.36 (1.22–1.53)	<0.001	1.13 (1.06–1.20)	<0.001
2	1.10 (0.91–1.33)	0.3	1.30 (1.08–1.56)	0.006	1.28 (1.17–1.40)	<0.001
>3	1.68 (1.33–2.12)	<0.001	1.94 (1.54–2.43)	<0.001	1.56 (1.38–1.77)	<0.001
Drug-resistant status						
Drug-susceptible	—		—		—	
Drug-resistant	2.34 (1.99–2.75)	<0.001	2.41 (2.06–2.81)	<0.001	1.61 (1.46–1.77)	<0.001
HIV status during the episode						
HIV-negative	—		—		—	
HIV-positive, not on ART	8.87 (7.55–10.4)	<0.001	7.42 (6.35–8.67)	<0.001	6.89 (6.31–7.52)	<0.001
HIV-positive, on ART	2.52 (2.21–2.86)	<0.001	2.12 (1.88–2.39)	<0.001	1.94 (1.83–2.06)	<0.001
HIV status unknown	1.14 (0.96–1.37)	0.14	1.20 (1.00–1.43)	0.045	1.19 (1.10–1.28)	<0.001
COVID-19 during episode						
No infection	—		—		—	
Infection	0.33 (0.21–0.53)	<0.001	0.41 (0.26–0.66)	<0.001	1.22 (1.06–1.39)	0.004
Diabetes						
Negative	—		—		—	
Positive	1.25 (1.04–1.50)	0.018	1.31 (1.10–1.57)	0.002	1.54 (1.42–1.67)	<0.001

* Adjusted for the month of first evidence and health district. Deaths grouped by TB first evidence date.

aOR = adjusted OR; CI = confidence interval; OR = odds ratio; ART = antiretroviral therapy.

The aOR for death during TB-specific admission was similarly higher with PHSM Levels 1–4 compared with pre-pandemic, with no difference during Level 5. The aOR for death vs. pre-pandemic was similar for Level 1 (1.4, 95% CI 1.2–1.6), Level 2 (1.4, 95% CI 1.2–1.8) and Level 4 (1.3,95% CI 1–1.4). Other factors associated with death were having more previous TB episodes, drug-resistant TB (aOR 2.3, 95% CI 2–2.8 vs. drug-susceptible), and living with HIV but not on ART (aOR 8.9, 95% CI 7.6–10.4 vs. no HIV).

The probability of death during TB-specific admission of PLHIV not on ART increased across all PHSM alert levels (Level 5, mean 0.5, 89% percentile interval 0.4–0.6) relative to before the advent of COVID-19 (mean 0.4, 89% percentile interval 0.3–0.4). The difference between the probability of death of PLHIV not on ART compared with people without HIV increased during some PHSM alert levels compared with before the COVID-19 pandemic (mean 0.3, 89% percentile interval 0.2–0.3), especially during Level 5 (mean 0.4, 89% percentile interval 0.3–0.5), but less so during the later less restrictive periods.

## DISCUSSION

We found an average 22% reduction (95% CI 19–25) in monthly TB episodes among adults in the public health sector in the Western Cape from the onset of COVID-19 compared with the expected counterfactual counts. The reduction over time in the impact of heightened restrictions on TB episode counts is likely due to reduced adherence to PHSM, increased health service usage, and efforts to maintain episode diagnosis.

Besides the brief period of Level 5 implementation, TB-specific admission mortality risk increased between 18% (95% CI 1–37) and 44% (95% CI 16–79) compared with pre-pandemic levels across different restriction periods. There are similar findings for all-cause mortality, although they are less marked. Furthermore, the risk of death increased as alert levels were relaxed from higher restrictions, with the risk of death during TB-specific admissions being the highest during Levels 1 and 2. Level 5 was in place at the start of the epidemic for 35 days, possibly explaining the non-significant estimates during that level and likely contributing to diagnosis delays in later episodes.

These findings suggest that reduced TB diagnoses during the most restrictive periods may have led to delayed diagnosis or limited diagnosis to individuals with more severe disease, increasing the mortality risk among diagnosed cases.

Historically, viral pandemics often coincide with an increased risk of TB deaths.^2^ This could result from service disruptions, interactions between infectious agents, or lung damage from concurrent or subsequent infections.^2^ Evidence shows increased mortality risk for individuals with concurrent or past TB and SARS-CoV-2 infections and that the COVID-19 pandemic response widely impacted TB services in both high- and low-resource settings.^8,9,16–19^ To assess the impact of service disruption, we focused on outcomes among individuals without evidence of COVID-19 at the time of outcome. Given extensive COVID-19 testing during admissions and recommended testing for those deceased with an unknown cause, we believe that our results are not directly influenced by COVID-19 on TB outcomes. Nonetheless, we could not fully assess the impact of all SARS-CoV-2 infections during or before a TB episode because there was likely substantial under-ascertainment of infections that occurred outside of hospitalisation.

Although we cannot measure when each TB episode would have been diagnosed without service disruptions or assess disease severity at diagnosis, our findings suggest that stricter PHSM alert levels caused delays and restrictions in TB diagnosis. This likely led to decreased TB episodes during the COVID-19 pandemic, with episode counts varying between high and low PHSM alert levels. These delays may be due to reduced mobility, changes in health-seeking behaviour, and increased pressure on health services that reduce TB screening and diagnosis. Thus, it is plausible that fewer TB-infected people presented for care and that those who did present were sicker because of delayed diagnosis, with a resultant increase in the risk of death among those diagnosed. Of concern is that at the end of the study period, the interrupted time series model showed that the cumulative difference between expected counterfactual TB episodes in the absence of the COVID-19 pandemic and actual TB episodes was still statistically significant despite the increased number of episodes diagnosed later in the pandemic. Therefore, the effects of restriction and delays are ongoing.

Increased TB mortality risk may also stem from disruptions in the management of comorbidities among adults with TB. Although ART provision remained robust in South Africa ^20^ and sub-Saharan Africa,^21^ there was a reduction in ART initiation across South Africa during 2020.^22^ We cannot separate the disruption of TB control from HIV treatment. Still, people with TB often have complex comorbidities, making them more susceptible to severe outcomes when diagnosis and treatment are delayed.

The limitations of this study include under-ascertainment of SARS-CoV-2 infections across TB episodes and uncertainty regarding the reason for admission. To address potential bias, the primary outcome of interest was deaths during TB-specific admission with a maximum follow-up of 6 months after the first evidence date of TB.

## CONCLUSION

TB control measures appear to have undergone more disruption globally than HIV control measures, which may indicate less robust health systems with less investment in low-resource settings.^23,24^ Managing new TB diagnoses and treatment initiation requires direct health service contact amidst pandemic service pressure and mobility-related restrictions. Recent models predict a global increase in TB deaths of 4% to 16%^25^ and 20%^26^ over the next 5 years, depending on the level of disruptions in TB control. This study shows a decrease in the number of TB episodes diagnosed and an increased risk of severe outcomes during the COVID-19 pandemic in 2020 and 2021. The recovery of TB programmes in low-resource settings is urgent,^8,10^ and TB management programmes must be integrated into future pandemic responses.
